# Monitoring of leucine‐rich alpha‐2‐glycoprotein and assessment by small bowel capsule endoscopy are prognostic for Crohn's disease patients

**DOI:** 10.1002/jgh3.12964

**Published:** 2023-09-05

**Authors:** Takahiro Ito, Kazuki Dai, Masashi Horiuchi, Toshiki Horii, Shigeru Furukawa, Atsuo Maemoto

**Affiliations:** ^1^ IBD Center Sapporo Higashi Tokushukai Hospital Sapporo Japan

**Keywords:** capsule endoscopy Crohn's disease activity index, Crohn's disease, leucine‐rich alpha‐2 glycoprotein, prognosis, small bowel capsule endoscopy

## Abstract

**Background and Aim:**

Endoscopy is important to determine the effectiveness of treatment for Crohn's disease (CD), but searching the entire small intestine is difficult. Thus, we investigated the usefulness of leucine‐rich alpha‐2 glycoprotein (LRG), a new biomarker for predicting mucosal activity, in evaluating the activity of CD small intestinal lesions. This will further determine whether the results of small bowel capsule endoscopy (SBCE) affect the prognosis of patients with CD.

**Methods:**

A total of 114 patients with CD who underwent SBCE were included. We analyzed the correlation between LRG and Capsule Endoscopy Crohn's Disease Activity Index (CECDAI). The cutoff value of LRG to achieve mucosal healing was calculated using the receiver operating characteristic curve. Then, we compared the presence or absence of intervention and the relapse rate of patients who could not achieve mucosal healing.

**Results:**

The CECDAI correlated with LRG. The calculated LRG value for achieving mucosal healing was ≤11.9. Ninety‐one patients were in clinical remission at the time of SBCE. During the follow‐up period, 17 patients relapsed. As a result of SBCE, when no treatment intervention was performed in the case of CECDAI ≥3.5, the relapse rate was significantly higher than when CECDAI <3.5 or intervention was performed in the case of CECDAI ≥3.5.

**Conclusions:**

The results reveal that LRG correlates with the activity of the entire small intestine and that SBCE assessment and therapeutic intervention can influence patient prognosis.

## Graphical abstract

As a result of small bowel capsule endoscopy, when no treatment intervention was performed in the case of Capsule Endoscopy Crohn's Disease Activity Index (CECDAI) ≥ 3.5, the relapse rate was significantly higher than when CECDAI < 3.5 or intervention was performed in the case of CECDAI ≥ 3.5.

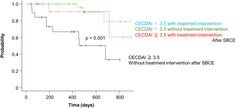

## Introduction

In Crohn's disease (CD), the accumulation of chronic inflammation causes intestinal adverse events, such as stenosis and abscess. Surgery is performed when intestinal dysfunction is unavoidable. Even after the surgery, anastomotic recurrence is high, and if the surgery is repeated for the small intestine, a short bowel syndrome may develop.

To improve the long‐term prognosis of patients with CD, it is necessary to start anti‐inflammatory treatment, including biologics, such as antitumor necrosis factor (TNF) agents, at an early stage to prevent intestinal complications. Confirmation of mucosal healing by endoscopic evaluation is indispensable for determining the therapeutic effect. Insertion to the terminal ileum by conventional ileocolonoscopy is not sufficient to confirm small intestinal lesions of CD; therefore, balloon‐assisted endoscopy (BAE) or small bowel capsule endoscopy (SBCE) is required.

While BAE allows biopsy and detailed evaluation of the mucosa, it is somewhat invasive, and observing the entire small intestine for CD lesions is difficult, which has many adhesions and deformities. On the contrary, SBCE is a noninvasive and simple examination that allows safe observation of the entire small intestine even in CD without severe stenosis. The degree of inflammation can be evaluated by SBCE using the Lewis score^1^ or the Capsule Endoscopy Crohn's Disease Activity Index (CECDAI).^2^ Although values are set to determine mucosal healing, the Lewis score is not originally a score specialized for CD evaluation. In addition, investigating whether CECDAI is also useful for predicting relapse of CD in remission is insufficient.

SBCE is challenging to perform frequently because of cost and examination time; therefore, the establishment of biomarkers that can easily predict endoscopic activity is important. Generally, C‐reactive protein (CRP) that reflects inflammation often does not increase even with the worsening of small intestinal lesions. Fecal calprotectin is an established marker of mucosal inflammation. Reports exist of a correlation between elevated calprotectin levels in patients with CD and inflammation in the colon, but none have been established for the small intestines. Therefore, developing a biomarker that more accurately correlates with the endoscopic activity of CD has been desired.

Leucine‐rich alpha‐2 glycoprotein (LRG), which was approved in Japan since June 2020, is attracting attention as a serum biomarker that can predict endoscopic activity in patients with inflammatory bowel disease (IBD).^3^ CRP is a protein that responds to interleukin (IL‐6) in the blood and is produced in the liver. On the contrary, LRG is a glycoprotein with a leucine‐rich repeat induced by various inflammatory cytokines, such as TNFα, IL‐12, and IL‐23, and a protein that is produced directly from the intestinal mucosa at the inflammation site and released into the blood.^4^ LRG is a novel serum biomarker for monitoring disease activity during therapy in patients with an autoimmune condition, which is particularly useful in patients with active disease but normal CRP levels. A correlation was reported between LRG and endoscopic activity in ulcerative colitis, and low LRG can be inferred to indicate mucosal healing.^3^

In CD, LRG and endoscopic activity measured by Simple Endoscopic Score for Crohn's disease (SES‐CD^5^) are also correlated, and it is considered that LRG is more useful than CRP and fecal calprotectin in predicting endoscopic activity. However, since SES‐CD is evaluated within the scope of conventional colonoscopy, it is unclear whether LRG is useful for predicting small intestinal lesions in CD. Kawamoto et al.^6^ performed extensive small intestinal observations using BAE in patients with CD and reported a correlation between the endoscopic activity of the small intestine (modified SES‐CD) and LRG, but not the entire small intestine. Kawamura et al.^7^ showed a correlation between LRG and endoscopic activity of the small intestine using BAE and reported the cutoff value of LRG, which predicted endoscopic remission, as 8.9 μg/mL. The number of patients in each case was small, and there is no established cutoff value of LRG to predict mucosal healing in the small intestine. It is also unclear whether LRG predicts subsequent prognosis. Therefore, this study aimed to calculate the cutoff value of LRG that could predict mucosal healing of the small intestine using SBCE and examine whether the score used in SBCE reflects the prognosis of patients with CD in remission and determine how the LRG should be used in daily practice.

## Methods

### Study design and population

This was an observational, retrospective single‐center study (IBD center, Sapporo Higashi Tokushukai Hospital, Hokkaido, Japan). We identified 165 patients with CD who underwent SBCE from July 2020 to September 2022. Patients with active lesions in the large intestine detected in the most recent ileocolonoscopy and with anal fistula were excluded because we wanted to focus only on the small intestine. Furthermore, patients in whom the entire small intestine could not be observed using SBCE were excluded.

The research protocol for this analysis was approved by the ethics committee of Tokushukai Group (Ref. no. TGE01388‐012) through the opt‐out method. The requirement for acquiring informed consent from patients was waived owing to the retrospective nature of this study.

### Data collection and outcomes

The baseline characteristics at the time of SBCE collected from the medical records were age, sex, disease duration, body mass index, disease location, disease behavior according to the Montreal Classification system, history of CD surgery, previous and concomitant CD medication (including enteral nutrition, budesonide, immunomodulators, and biotherapy), CDAI score,^8^ white blood cell count (WBC, cells/μL), hemoglobin concentration (g/dL), platelet count (×10^4^/μL), CRP (mg/dL) level, serum albumin level (g/dL), and LRG (μg/mL).

We used PillCam SB3 (Medtronic, MN, Minneapolis, USA) in all SBCE examinations. SBCE images were assessed by three physicians certified by the Japanese Association for Capsule Endoscopy with more than 10 years of experience. The Lewis score^1^ and CECDAI^2^ were used to evaluate endoscopic activity in SBCE. We analyzed the correlation between the WBC, CRP, and LRG and the Lewis score or CECDAI. Then, we compared the presence or absence of intervention and the relapse rate of patients who could not achieve mucosal healing. The need for therapeutic intervention was determined by the attending physician based on the results of the SBCE. Relapse was defined as a worsening of CDAI above 151 or the need for therapeutic intervention.

### Statistical methods

Continuous variables are presented as percentages or medians with min–max ranges. Spearman's rank correlation coefficient was used to analyze the correlations between the CECDAI or Lewis score and WBC, CRP, and LRG. ROC curve analysis was used to identify the optimal cutoff values of LRG with maximum sensitivity and specificity for achieving mucosal healing (CECDAI <3.5 or Lewis score <135^1^, ^2^) and the area under the curve (AUC) was obtained. The Kaplan–Meier method was used to assess whether the relapse rate differs depending on the presence or absence of intervention. Significant differences between outcomes were indicated by *P* values <0.05. All statistical analyses were performed with EZR (Saitama Medical Center, Jichi Medical University, Saitama, Japan), which is a graphical user interface for R (The R Foundation for Statistical Computing, Vienna, Austria).

## Results

### Baseline characteristics

A total of 114 patients were included in this retrospective cohort study. The baseline characteristics of the included patients are present in Table 1. Of the patients, 41.2% (*n* = 47/114) had an ileal disease (L1), 52.6% (*n* = 60/114) had an ileocolonic disease (L3), and 6.1% (*n* = 7/114) had a colonic disease (L2), but they did not have inflamed large intestine when performing SBCE. We did not exclude L2 because it is considered necessary to assess the small bowel, as new small bowel lesions may be detected after a longer duration of disease, even if the patient originally had L2. Seventy‐three patients underwent patency capsule (PC) tests, which were useful for confirming the patency of the small bowel. Patients who have performed SBCE every year or whose CT or gastrointestinal radiography within the past year has not revealed severe stenosis may be omitted from the PC tests after explaining this to the patients.

**Table 1:** Baseline characteristics

Baseline characteristics	N (%) or median (min–max)
Age	35.0 (16–71)
Sex (male)	83 (72.8)
Disease duration, months	141.7 (2.2–475.9)
BMI	21.2 (16.6–32.2)
Disease location	
Ileum (L1)	47 (41.2)
Colon (L2) *No inflammation of the large intestine when performing SBCE	7 (6.1)
Ileocolonic (L3)	60 (52.6)
Disease behavior	
Inflammatory disease (B1)	50 (43.9)
Stricturing disease (B2)	31 (27.2)
Penetrating disease (B3)	33 (28.9)
Prior small intestinal resections	56 (49.1)
Stoma	17 (14.9)
Disease activity	
CDAI score	40 (0–204)
White blood cell, /μL	5330 (1080–11 350)
Hemoglobin, g/dL	14.4 (8.8–17.0)
Platelet, ×10^4^/μL	23.6 (5.9–50.5)
Albumin, g/dL	4.5 (2.9–5.4)
CRP, mg/dL	0.04 (0.01–6.77)
LRG, μg/dL	11.5 (6.6–30.0)
Endoscopic activity of SBCE	
Lewis score	174 (0–4560)
CECDAI score	3 (0–17)
Treatment at the time of SBCE	
Enteral nutrition	44 (38.6)
Budesonide	5 (4.4)
Immunomodulators	46 (40.4)
Biologics	90 (78.9)

### Correlation between endoscopic scores and biomarkers

**Figure 1:**
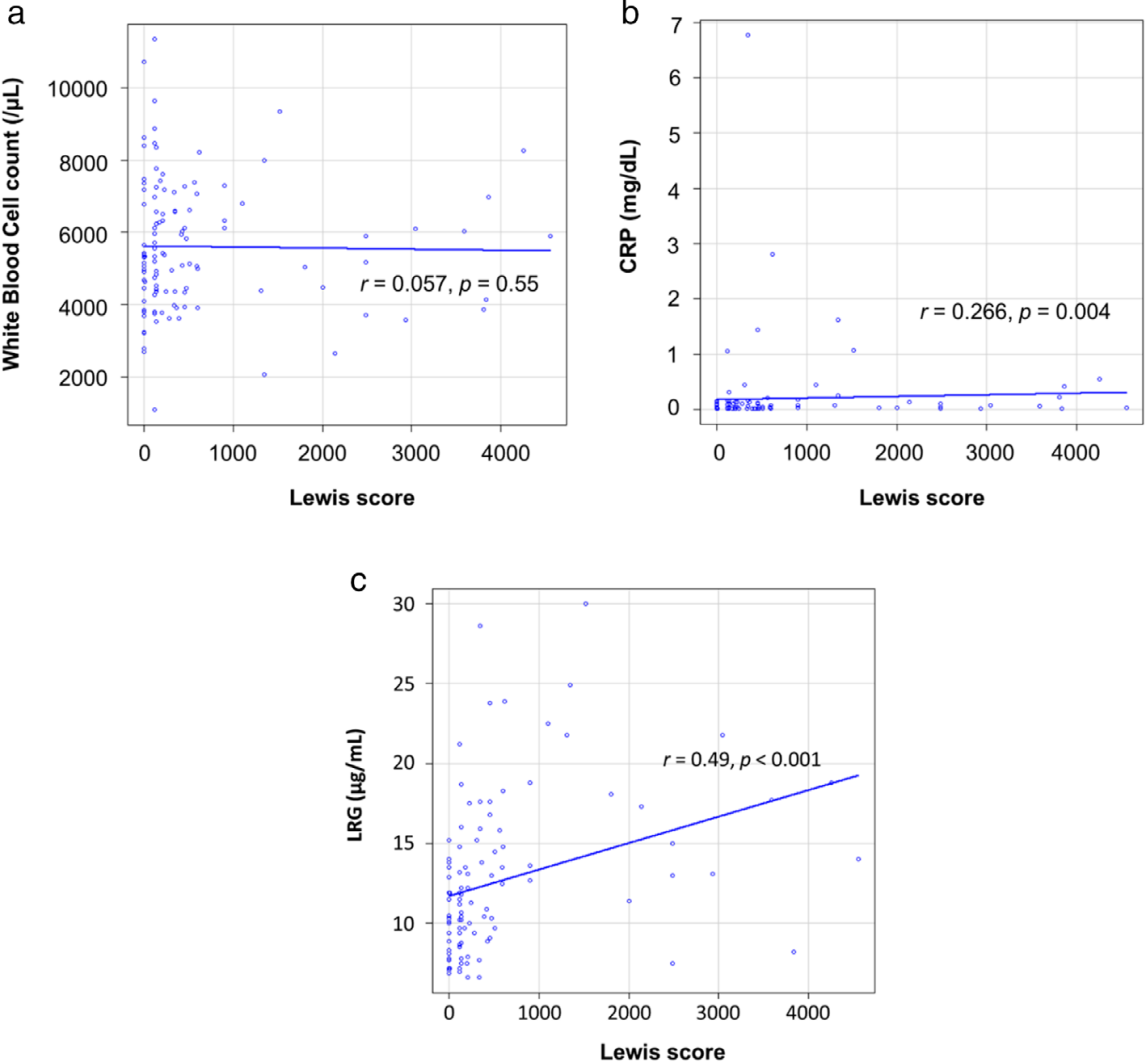
Correlation between Lewis score and biomarkers. The relationship between the Lewis score and biomarkers was examined using a correlation chart. All serial time points were put into the chart. The Lewis score ranged from 0 to 4560. No correlation was found between the Lewis score and the WBC count (a). A weak correlation was found between the Lewis score and CRP (b). The Lewis score correlated with LRG (c).

**Figure 2:**
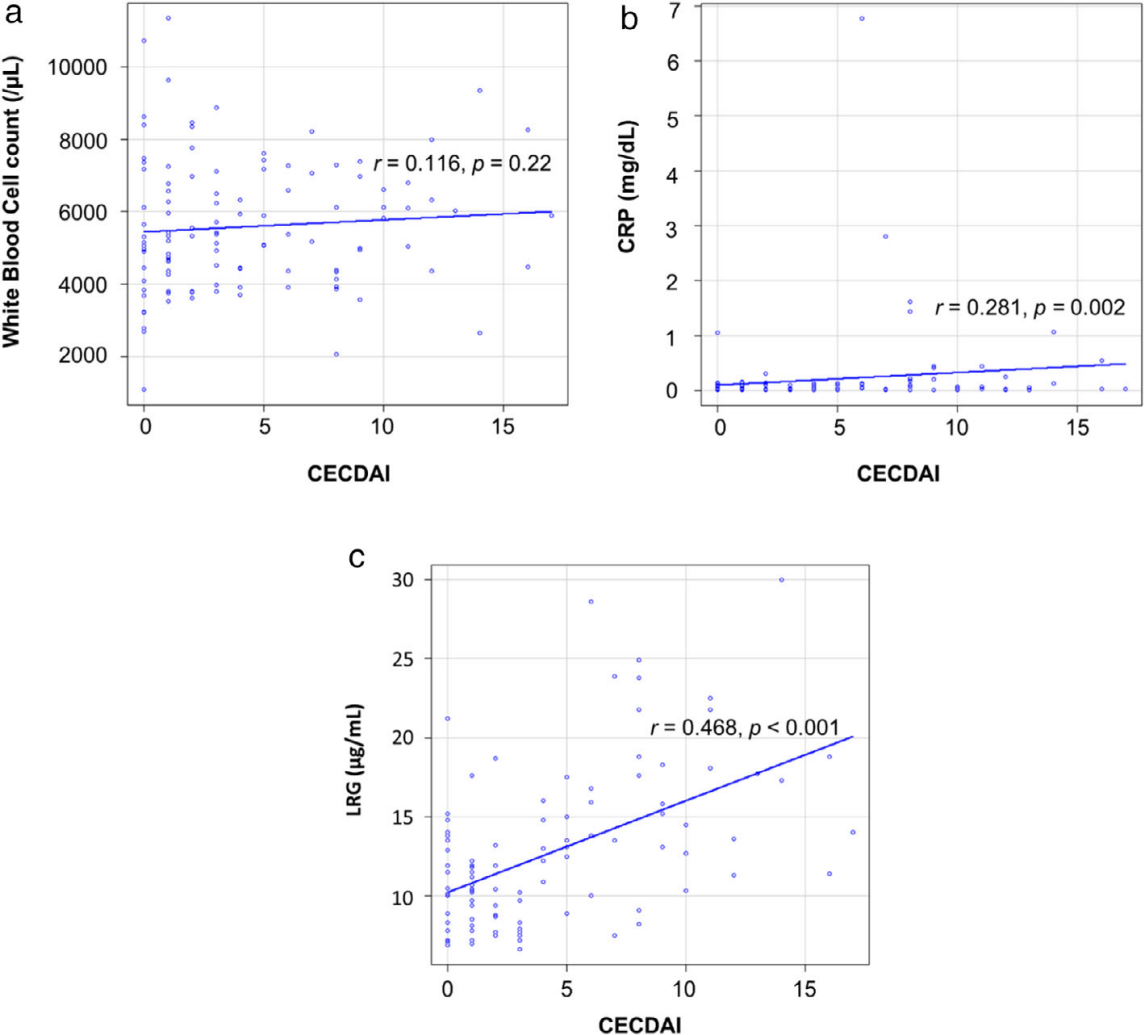
Correlation between CECDAI and biomarkers. The relationship between the CECDAI and biomarkers was examined using a correlation chart. All serial time points were added to the chart. The CECDAI ranged from 0 to 17. No correlation was found between CECDAI and the WBC count (a). A weak correlation was found between CECDAI and CRP (b). The CECDAI correlated with LRG (c).

The relationship between endoscopic scores and biomarkers was examined using a correlation chart. No correlation was found between the Lewis score and the WBC count (*r* = 0.057) (Fig. 1a). A weak correlation was found between the Lewis score and CRP (*r* = 0.266) (Fig. 1b). The Lewis score was correlated with LRG (*r* = 0.49) (Fig. 1c). No correlation was found between CECDAI and the WBC count (*r* = 0.116) (Fig. 2a). A weak correlation was found between CECDAI and CRP (*r* = 0.281) (Fig. 2b). CECDAI was correlated with LRG (*r* = 0.468) (Fig. 2c). LRG showed the strongest correlation with the Lewis score and CECDAI.

### Determination of LRG values to predict mucosal healing

**Figure 3:**
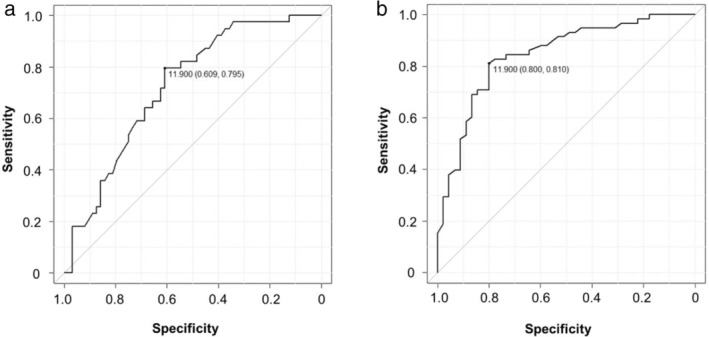
Determination of LRG values to predict mucosal healing. ROC curve analysis was used to identify optimal cutoff values of LRG with maximum sensitivity and specificity for achieving mucosal healing (Lewis score <135 or CECDAI <3.5). In ROC curve analysis, (a) the calculated LRG value for achieving the “Lewis score” of <135 was ≤11.9, and the AUC was 0.723; sensitivity, 79.5%; specificity, 60.9%; PPV, 55.4%; NPV, 83.0%; and accuracy, 68.0%. (b) The calculated LRG value for achieving CECDAI of <3.5 was also ≤11.9, and the AUC was 0.843; sensitivity, 81.0%; specificity, 80.0%; PPV, 83.9%; NPV, 76.6%; and accuracy, 80.6%.

We considered using LRG to estimate mucosal healing of the small intestine. The calculated LRG value for achieving the Lewis score of <135 was ≤11.9, and the AUC was 0.723; sensitivity, 79.5%; specificity, 60.9%; positive‐predictive value (PPV), 55.4%; negative‐predictive value (NPV), 83.0%; and accuracy, 68.0% (Fig. 3a). The calculated LRG value for achieving CECDAI<3.5 was also ≤11.9, and the AUC was 0.843 (sensitivity, 81.0%; specificity, 80.0%; PPV, 83.9%; NPV, 76.6%; and accuracy, 80.6%) (Fig. 3b).

### Treatment intervention after SBCE prevents relapse

Ninety‐one patients were in remission (CDAI ≤150) at the time of performing SBCE. Patients with stomas were excluded because the number of diarrhea could not be counted and the CDAI could not be calculated. Routine SBCE is performed even when symptoms are in remission to determine the response to the treatment or to determine whether the patient is endoscopically active.

In the present study, all patients did not have active lesions in the large intestine and anal fistula; thus, the decision to intervene or follow‐up treatment was made after discussion between the attending physician and the patient based on the symptoms and the activity in the small bowel. No patient had intensified treatment due to colorectal or anal disease.

The relationship between treatment intervention after SBCE and relapse was investigated using the Kaplan–Meier method. Treatment interventions include switching biologics, increasing the dose of biologics, or adding budesonides or immunomodulators. During the follow‐up period, 17 of 91 patients had relapsed.

**Figure 4:**
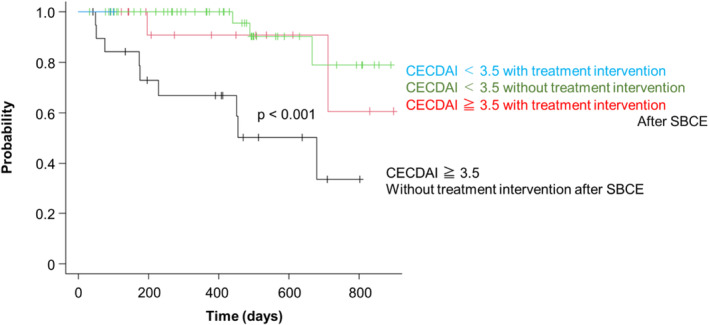
Relationship between treatment intervention and relapse according to SBCE results. The relationship between treatment intervention after SBCE and relapse was investigated using the Kaplan–Meier method. As a result of SBCE, when no treatment intervention was performed in the case of CECDAI ≧ 3.5 (black), the relapse rate was significantly higher than when intervention was performed in the case of CECDAI ≧ 3.5 or CECDAI <3.5 (*P* < 0.001).

As a result of SBCE, when no treatment intervention was performed in the case of CECDAI ≧ 3.5, the relapse was significantly higher than when CECDAI <3.5 or intervention was performed in the case of CECDAI ≧ 3.5 (*P* < 0.001) (Fig. 4). The median CECDAI score for patients with CECDAI ≧3.5 was 8 (min–max, 4–17).

**Figure 5:**
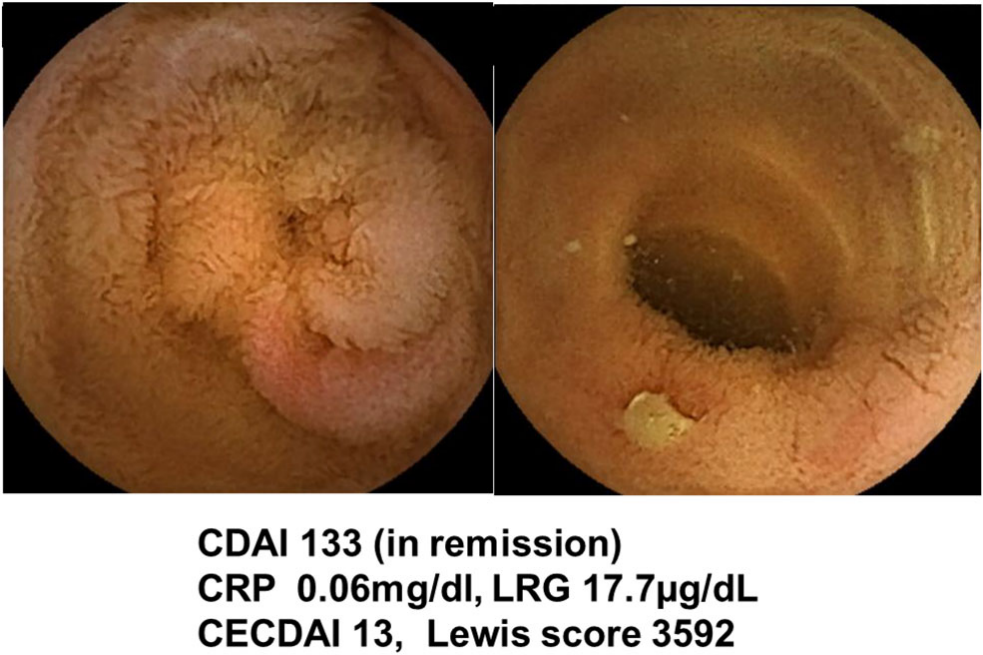
Images of SBCE in a patient with CD in clinical remission. Multiple small ulcers and erosions are seen in the entire small intestine. This male patient in his 70s had the ileitis type, and he received vedolizumab. His CRP level was within the normal range, and his LRG level was mildly elevated. The CECDAI was 13, and the Lewis score was 3592, but no additional treatment was given at the patient's request. His symptoms flared up 51 days after the SBCE.

Figure 5 shows a case in which SBCE was performed on a patient with CD in remission. This male patient in his 70s had ileal CD and was treated with vedolizumab. His CRP was within the normal range, and the LRG level was mildly elevated. His CECDAI and Lewis score were 13 and 3592, respectively, but no additional treatment was given at the patient's request. His symptoms flared up 51 days after the SBCE.

## Discussion

The calculated LRG value for achieving mucosal healing defined by CECDAI was ≤11.9. Among 91 patients in clinical remission, 17relapsed. As a result of SBCE, when no treatment intervention was performed in the case of CECDAI ≥3.5, the relapse rate was significantly higher than when CECDAI <3.5 or when intervention was performed in the case of CECDAI ≥3.5. Although not limited to CD, several methods of examining the small intestine are available, including small bowel barium enteroclysis (SBE), BAE, CT enterography (CTE), magnetic resonance enterography (MRE), ultrasonography (US), and SBCE. Each examination has both advantages and disadvantages and should be selected according to the purpose of the examination and the need for treatment.

SBE is characterized by its ease of evaluation of fine mucosal surfaces, but has the problem of radiation exposure.^9^, ^10^ BAE is the best at evaluating mucosal surfaces, but it is associated with radiation exposure and has occasional complications, and BAE insertion requires skill. In addition, observing the entire small intestine is difficult because of intestinal deformity and adhesions.^10^ CTE and MRE are good at capturing transmural inflammation and extraintestinal changes, but are not at detailed evaluation of mucosal surface lesions.^9^, ^10^ Although US is a noninvasive and easy examination, it is not widely performed for CD because the results are dependent on the proficiency of the operator.^10^ SBCE is a simple and easy examination with no radiation exposure and is good at evaluating mucosal surfaces, but it cannot be performed in the presence of severe intestinal stenosis because of retention.^10^

In CD, evaluating the small intestine by conventional ileocolonoscopy alone is not sufficient, and there have been increasing reports that SBCE is useful in the search for small intestinal lesions in CD.^11^ SBCE is also reported to be useful in detecting and treating jejunal lesions that could not be detected by other examination methods.^12^, ^13^ The degree of inflammation can be evaluated by SBCE using the Lewis score^1^ or CECDAI.^2^ In this study, the sensitivity, specificity, and positive‐predictive value of LRG for predicting mucosal healing may have differed because the Lewis score is not specific to CD and the CECDAI is a score developed for CD. It is interesting to note that when using LS <135 or CECDAI<3.5 to obtain a cutoff value of the LRG predicting mucosal healing, both results had the same LRG≤11.9. These results suggest that LS could also be useful in predicting prognosis.

Nishikawa et al. reported that the Lewis score is useful in the evaluation of small intestinal lesions in CD.^14^ They analyzed the association of the Lewis score with the prognosis of 86 patients who did not receive therapeutic intervention after SBCE. It was observed that subsequent hospitalization and relapse rates were lower when the Lewis score was <264. On the contrary, the prognosis of the treatment group is not described. Omori et al. also reported a correlation between LRG and endoscopic activity of the small intestine using SBCE, but they did not investigate the course of symptoms after performing SBCE.^15^ Miyazu et al. reported the association between severities classified by CECDAI and treatment intervention, but did not investigate the course of symptoms after treatment change.^16^ Elosua et al. reported that 51.3% of patients changed treatment after 432 SBCE examinations and that SBCE was effective.^17^ However, this report did not investigate the course of symptoms after the change in treatment. To our knowledge, we showed for the first time the relationship between treatment intervention and prognosis based on SBCE results. In other words, the prognosis was better when treatment intervention was performed when CECDAI was ≥3.5.

Miyazu et al. reported that CECDAI and Lewis score did not correlate with biomarkers such as albumin, hemoglobin, and CRP.^16^ This indicates that existing biomarkers cannot predict the inflammatory activity in the mucosa of the small intestine. LRG is attracting attention as a serum biomarker that can predict endoscopic activity in patients with IBD.^3^ A correlation was found between LRG and endoscopic activity in ulcerative colitis, and low LRG can be inferred to indicate mucosal healing.^3^

**Figure 6:**
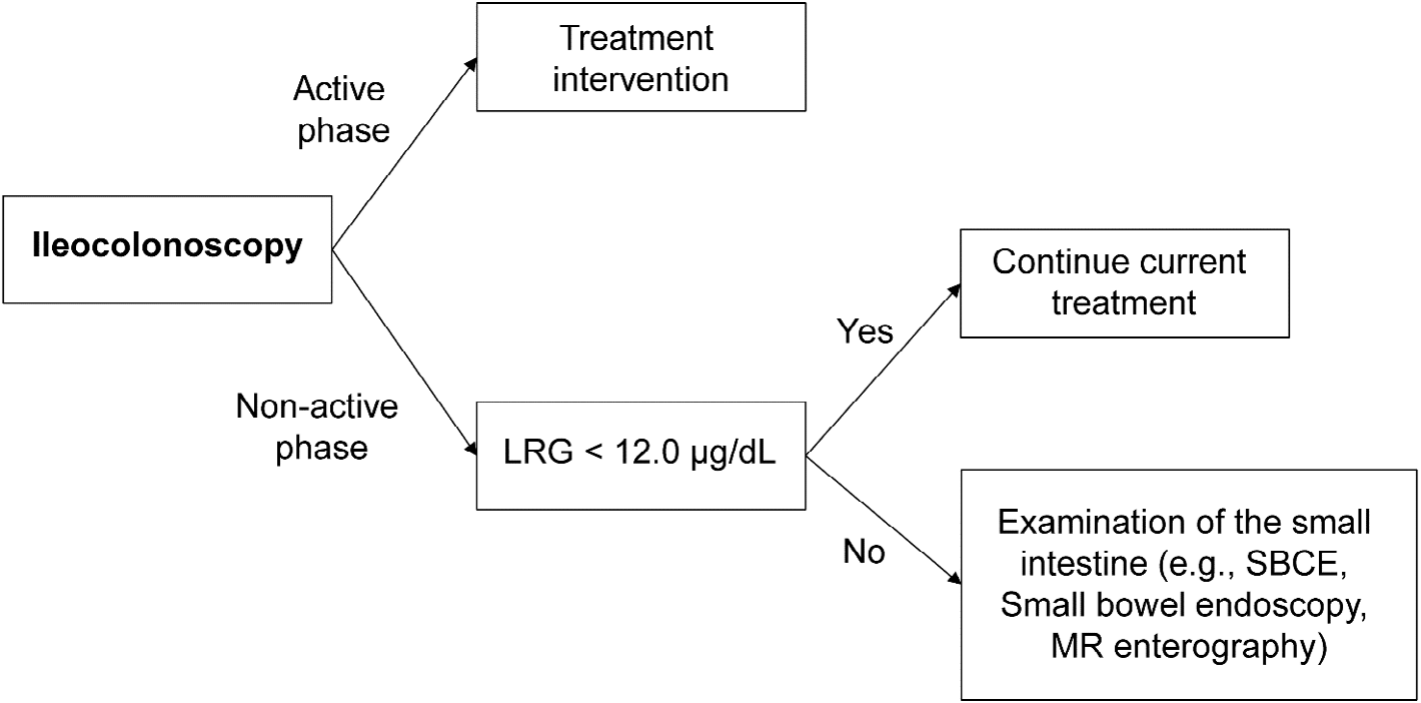
Flow of examination with reference to LRG values. When a patient with Crohn's disease does not have active lesions during colonoscopy, if the LRG is ≤12 μg/dL, an aggressive small bowel examination using SBCE or other methods is recommended, and if the activity is high, performing therapeutic intervention is advisable.

As mentioned for CD, a correlation was found between endoscopic activity and LRG within the range observable by conventional colonoscopy and small bowel endoscopy. However, a few studies have evaluated the entire small intestine. Thus far, CRP levels have not been elevated in patients with CD with worsening small intestinal lesions, often resulting in delayed therapeutic intervention. The response of newly developed LRG to small intestinal lesions in CD was also unknown. This study demonstrated that the LRG is useful in the evaluation of the activity of small intestinal lesions in CD. The LRG of ≤11.9 is predictive of mucosal healing of small intestinal lesions in CD. The study also showed that the prognosis is better if therapeutic intervention is performed when the CECDAI is ≥3.5, even if symptoms are in remission. Therefore, even if a patient with CD does not have active lesions during ileocolonoscopy, if the LRG is ≥12 μg/dL, an aggressive small bowel examination using SBCE or other methods is recommended, and if the activity is high, therapeutic intervention may be necessary (Fig. 6). Of course, even if the terminal ileum is involved in ileocolonoscopy, the entire small intestine should be observed whenever possible. It is important that in cases of CECDAI ≥3.5, the attending physician and the patient should thoroughly discuss the need for therapeutic intervention and what to do if symptoms worsen.

This study has some limitations. First, this study has a retrospective single‐center design. Second, although the correlation values of the LS and CECDAI in the previous report (135 and 3.5) are used, there is no guarantee that these two scores will show the same correlation values as in the previous report in the target population of this study. Third, SBCE images were assessed by three physicians certified by the Japanese Association for Capsule Endoscopy with more than 10 years of experience, but the variability of the results was not analyzed. After each of the three physicians assessed the SBCE images of their own patients using CECDAI and Lewis scores, the final decision was made centrally by me. Fourth, few patients have had fecal calprotectin (a biomarker of Crohn's disease activity) measured, without confirming its correlation with LRG or the superiority of LRG. Fifth, the presence or absence and content of therapeutic intervention after SBCE are dependent on the discretion of each physician. Prospective randomized controlled trials should be conducted in the future to verify whether LRG values and SBCE results improve the prognosis of patients with CD.

To our knowledge, this is the first study to show that LRG correlates with the activity of the entire small intestine and that SBCE and therapeutic intervention can influence the prognosis of patients with CD.

## Data Availability

Access to the data supporting the results of this study will be requested and reviewed with the principal investigator of this study through the corresponding author. The data are not available to the public due to privacy and ethical restrictions.

## References

[1] Gralnek IM , Defranchis R , Seidman E , Leighton JA , Legnani P , Lewis BS . Development of a capsule endoscopy scoring index for small bowel mucosal inflammatory change. Aliment. Pharmacol. Ther. 2008; 27: 146–154. 17956598 10.1111/j.1365-2036.2007.03556.x

[2] Gal E , Geller A , Fraser G , Levi Z , Niv Y . Assessment and validation of the new capsule endoscopy Crohn's disease activity index (CECDAI). Dig. Dis. Sci. 2008; 53: 1933–1937. 18034304 10.1007/s10620-007-0084-y

[3] Shinzaki S , Matsuoka K , Iijima H *et al*. Leucine‐rich alpha‐2 glycoprotein is a serum biomarker of mucosal healing in ulcerative colitis. J. Crohns Colitis. 2017; 11: 84–91. 27466171 10.1093/ecco-jcc/jjw132 PMC5175492

[4] Serada S , Fujimoto M , Terabe F *et al*. Serum leucine‐rich alpha‐2 glycoprotein is a disease activity biomarker in ulcerative colitis. Inflamm. Bowel Dis. 2012; 18: 2169–2179. 22374925 10.1002/ibd.22936

[5] Daperno M , D'Haens G , Assche GV *et al*. Development and validation of a new, simplified endoscopic activity score for Crohn's disease: the SES‐CD. Gastrointest. Endosc. 2004; 60: 505–512. 15472670 10.1016/s0016-5107(04)01878-4

[6] Kawamoto A , Takenaka K , Hibiya S , Ohtsuka K , Okamoto R , Watanabe M . Serum leucine‐rich alpha2 glycoprotein: a novel biomarker for small bowel mucosal activity in Crohn's disease. Clin. Gastroenterol. Hepatol. 2022; 20: e1196–e1200. 34216822 10.1016/j.cgh.2021.06.036

[7] Kawamura T , Yamamura T , Nakamura M *et al*. Accuracy of serum leucine‐rich alpha‐2 glycoprotein in evaluating endoscopic disease activity in Crohn's disease. Inflamm. Bowel Dis. 2023; 29: 245–253. 35436345 10.1093/ibd/izac076

[8] Best WR , Becktel JM , Singleton JW , Kern F Jr . Development of a Crohn's disease activity index. National Cooperative Crohn's Disease Study. Gastroenterology. 1976; 70: 439–444. 1248701

[9] Bungay H . Small bowel imaging in Crohn's disease. Frontline Gastroenterol. 2012; 3: 39–46. 28839630 10.1136/flgastro-2011-100007 PMC5517243

[10] Saibeni S , Rondonotti E , Iozzelli A *et al*. Imaging of the small bowel in Crohn's disease: a review of old and new techniques. World J. Gastroenterol. 2007; 13: 3279–3287. 17659666 10.3748/wjg.v13.i24.3279 PMC4172707

[11] Sorrentino D , Nguyen VQ . Clinically significant small bowel Crohn's disease might only be detected by capsule endoscopy. Inflamm. Bowel Dis. 2018; 24: 1566–1574. 29893950 10.1093/ibd/izy048

[12] Tai FWD , Ellul P , Elosua A *et al*. Panenteric capsule endoscopy identifies proximal small bowel disease guiding upstaging and treatment intensification in Crohn's disease: a European multicentre observational cohort study. United European Gastroenterol J. 2021; 9: 248–255. 10.1177/2050640620948664 PMC8259365 32741315

[13] Le Berre C , Trang‐Poisson C , Bourreille A . Small bowel capsule endoscopy and treat‐to‐target in Crohn's disease: a systematic review. World J. Gastroenterol. 2019; 25: 4534–4554. 31496630 10.3748/wjg.v25.i31.4534 PMC6710184

[14] Nishikawa T , Nakamura M , Yamamura T *et al*. Lewis score on capsule endoscopy as a predictor of the risk for Crohn's disease‐related emergency hospitalization and clinical relapse in patients with small bowel Crohn's disease. Gastroenterol. Res. Pract. 2019; 2019: 4274257. 30944562 10.1155/2019/4274257 PMC6421745

[15] Omori T , Sasaki Y , Koroku M *et al*. Serum leucine‐rich alpha‐2 glycoprotein in quiescent Crohn's disease as a potential surrogate marker for small‐bowel ulceration detected by capsule endoscopy. J. Clin. Med. 2022; 11: 2494. 35566620 10.3390/jcm11092494 PMC9101788

[16] Miyazu T , Ishida N , Takano R *et al*. Usefulness of the capsule endoscopy Crohn's disease activity index in assessing the necessity of early additional treatment in patients with Crohn's disease in clinical remission. Medicine. 2021; 100: e26550. 34398010 10.1097/MD.0000000000026550 PMC8294877

[17] Elosua A , Rullan M , Rubio S *et al*. Does capsule endoscopy impact clinical management in established Crohn's disease? Dig. Liver Dis. 2022; 54: 118–124. 34518128 10.1016/j.dld.2021.08.014

